# Measuring users’ psychological self-withdrawal on mobile social media: the development of a context dependent instrument

**DOI:** 10.3389/fpsyg.2023.1296747

**Published:** 2024-01-05

**Authors:** Cheng Chen, Hong Yu

**Affiliations:** School of Business Administration and Tourism Management, Yunnan University, Kunming, Yunnan, China

**Keywords:** mobile social media use, psychological self-withdrawal, psychological self-withdrawal measure, WeChat context, mixed methods

## Abstract

**Introduction:**

MSM users have many concerns when disclosing information, such as role conflicts and privacy risks, which can result in nondisclosure. We call these negative feelings associated with MSM use “psychological self-withdrawal” (PSW).

**Methods:**

Using a mixed-methods design, this study develops and validates an instrument for measuring PSW in the WeChat context, based on focus-group interviews (seven participants), one-on-one interviews (eight participants), and a survey of 1200 WeChat users.

**Results:**

The result shows that PSW measure is a four-item instrument designed to measure PSW in the WeChat context. The results of two exploratory factor analysis pilot tests (*n* = 91 and *n* = 81) and a final confirmatory factor analysis test (*n* = 1200) indicate that the PSW measure has good internal consistency (*α* = 0.839), factorial validity, and a single-factor structure.

**Discussion:**

The study defines PSW as “users’ series of negative feelings when using WeChat” and develops and validates an PSW measurement, using focus groups, interviews, and a survey. A four-item PSW with acceptable reliability and validity was finally developed.

## 1 Introduction

Many users are now connected via mobile social media (MSM), which is defined as “a set of connectivity-enabled mobile applications that facilitate interaction and the co-creation, exchange, and publication of information” (Hoehle et al., 2015; Wong et al., 2022). The number of social media users has been on the rise and has reached one billion (Hew et al., 2019). With the increasing penetration of mobile phones, there is expected to be a more significant rise in MSM use in the future (Apaolaza et al., 2019).

However, increased use does not necessarily bring higher participation. Following its extraordinary growth in terms of use and diffusion, MSM has recently seen a decline in active users (Cao and Sun, 2018). Pressures such as social media exhaustion have led users to develop various coping behaviors, such as reducing posting frequency (You and Liu, 2022), using social media discontinuously (Fu et al., 2020), or deleting social media accounts (Lee, 2021), exhibiting certain forms of self-withdrawal (Ritchie, 2018). The phenomenon can be observed on many platforms. For example, Facebook, the world’s largest social networking site, has seen a shrinking user base in recent years (Cao and Sun, 2018). Similar to Facebook in the US, WeChat, China’s most popular social networking site (Chen et al., 2021), has also experienced a slowdown in user growth (Zhou and Tian, 2023).

Although some people still maintain their engagement on MSM, their use styles are often passive. Today, not all MSM users are willing to actively update their statuses, make comments, or engage in other forms of direct exchange. Many users just browse and observe content posted by others without any attempts to interact socially with other users (Liu et al., 2022). Such passive use behaviors involve features such as neglect and avoidance (Li et al., 2022). Viewed from the perspectives of the stressor–strain–outcome model, self-discrepancy theory, the affective response model, uses and gratifications theory, social cognitive theory, and the stimuli–organism–response framework, these passive use behaviors can be understood as motivated by negative psychological experiences, such as privacy concerns and social overload.

Passive use behavior can threaten the development of platform enterprises. It is commonly acknowledged that market value depends highly on benign interactions among active users (Carah and Shaul, 2016), and the decreased use of social networks could lead to financial losses for platform-based enterprises (Choi et al., 2015). This emphasizes the need for platform enterprises to better understand the concept of “psychological self-withdrawal” (PSW), which pertains to users’ uncomfortable feelings and passive engagement behaviors when using MSM.

A number of studies have focused on the SW phenomenon. From a user-behavior perspective, definitions and scales have been developed to understand and measure SW. These definitions have been widely used, and the measures have been validated, highlighting their suitability for measuring SW behavior in the MSM context. SW has been defined as the “active retention of information” (Dienlin and Metzger, 2016), which can be manifested in various ways, such as using privacy protection software or privacy settings (Meier et al., 2020), reducing public disclosure, or withholding certain information (Ritchie, 2018). However, according to stimuli–organism–response theory, behavior can be stimulated by psychological factors. Hence, existing definitions and measures of SW ignore the psychological perspective. Moreover, although researchers have found that users typically vacillate between self-disclosure and SW (Ma et al., 2021), neither the concept of PSW on MSM nor its measurement has been adequately addressed (Dienlin and Metzger, 2016), especially for acquaintance MSM platforms such as WeChat. This study, therefore, aims to fill gaps in MSM research by addressing the research questions that follow:

*RQ1*: How do we define psychological self-withdrawal on WeChat?

*RQ2*: How can we measure users’ psychological self-withdrawal on WeChat?

PSW on WeChat cannot be adequately examined without considering its connection to MSM use. Although there are many definitions of SW across different research areas and some measures of its construct, little has been done to define PSW and formalize its measurement in the MSM context, especially for WeChat, which is an acquaintance social networking platform. The few MSM researchers who have attempted to define SW often simply describe it as withholding information or deleting posts (e.g., Dienlin and Metzger, 2016; Chen, 2018), and the limited studies that attempted to measure SW often utilized scales with only physical-based items (e.g., Andreassen et al., 2012; Meier et al., 2020), ignoring SW’s psychological dimension. Thus, there is a need to further define SW in the psychological dimension and develop the related measurement items for the WeChat context. In this paper, we define PSW and develop a measurement instrument for PSW in the WeChat context using mixed methods from a psychology perspective instead of a behavior perspective. In the following, we first discuss MSM-related concepts and review SW research as the theoretical basis for developing the concept of PSW in the WeChat context. Second, we take PSW as a theoretical variable and develop its measurement items using a focus group with seven participants and interviews with eight WeChat users. Third, we use a study involving 1,200 WeChat users to empirically evaluate PSW. Lastly, we discuss the results, limitations, and future research directions.

## 2 Literature review

### 2.1 MSM and WeChat

According to Ju et al. (2021), MSM refers to “a set of platform-based media technologies that enable communication, interaction, and user-generated content creation and sharing,” and it encompasses two types of mobile applications: mobile social networking sites (e.g., Facebook and Twitter) and instant messaging apps (e.g., Facebook Messenger, WeChat, and WhatsApp). In order to adapt to the research context of this article, we define MSM as “a platform supported by a set of media technologies.” Thus, for this study, we regard WeChat as an MSM platform.

The difference between WeChat and other MSM applications is that WeChat is a strong-tie social platform (Chen et al., 2019) while applications such as Twitter are weak-tie social platforms (Valenzuela et al., 2018). Thus, WeChat has the characteristics of a social networking platform for acquaintances. In Twitter’s communication space, the “imagined audiences” can include strangers and even celebrities (Lee, 2013). While WeChat is more private and exclusive, more relationship focused, and less visible (Xu and Zhang, 2022), the imagined audiences can be friends, colleagues, and family members. WeChat has a relatively rigorous friending mechanism in which people can only add friends by scanning a private QR code or using a certain account ID, which means most WeChat friends are likely in some way connected offline (Pang, 2020). Most studies focus on weak-relationship MSM platforms such as Facebook and Twitter (Kim, 2023), ignoring strong-relationship MSMs such as WeChat (Ma et al., 2020). This is why we conducted this study based on the WeChat context.

WeChat (or *Weixin* in Chinese), launched in January 2011 by Tencent, is one of the most widely used MSMs among young people in China (Pang, 2020). It is an instant messaging and social networking platform that enables interactive exchange through mobile devices (Xu et al., 2015). People use WeChat to maintain or promote relations with acquaintances (Chen et al., 2021) by sending messages, making video or voice calls, and posting content (e.g., text, pictures, and videos) through “Moment,” exclusively to a single WeChat friend, or to a WeChat group. WeChat users can leave comments or simply click the “like” button on other users’ posts. It offers users a private platform on which only people admitted to one’s friend list can read one’s posts and comment on them (Pang, 2018). Thus, WeChat is considered a “must-have MSM app” in China (Zhang et al., 2021), and as of June 2022, it had 1.299 billion active users (Hong et al., 2023).

Given the characteristics of WeChat’s acquaintance network, users’ PSW might become more obvious on this platform. In the context of WeChat, people’s attitudes toward friends and their participation behavior can vary based on the strength of their *guanxi* network with other members (Shao and Pan, 2019). For example, users might disclose more freely to those they have strong ties with since they have long-established emotional attachments. Meanwhile, they might be more cautious about disclosing to those they have weak ties with since there is weaker trust. As the number of WeChat friends increases—especially the number of “weakly related” contacts—users begin to passively browse the “moments” of their contacts without actively interacting (Teng et al., 2022). Thus, MSM platform operators face the challenge of how to increase user activity and self-disclosure behavior (Ma et al., 2020) and reduce SW behavior.

### 2.2 Passive MSM use

Various environmental and social stimuli on MSM can produce different feelings in users, thus leading to different use styles. One dominant approach is to dichotomize MSM use into active and passive (Meier and Krause, 2022). Burke et al. (2010) were the first to propose this idea (Valkenburg et al., 2022). According to Yin et al. (2022), passive use is the most frequent pattern of MSM use.

Passive use refers to “monitoring the online life of other users without engaging in direct exchanges with them (e.g., scrolling through news feeds or looking at other users’ profiles)” (Verduyn et al., 2020). Passive users engage with MSM mainly to browse, monitor, and observe ongoing discussions (Dang, 2020) and to consume information.

Passive MSM use can lead to many negative outcomes. Many studies have found that passive social media use can be related to the feelings of envy (Kashian, 2022), low self-esteem, and other mental health issues; it can trigger an increased social comparison tendency (Ellison et al., 2020) and negatively affect well-being (Yue et al., 2022). If users believe their information on MSM is being recorded, leaked, or spread, they might adopt passive use behavior to protect personal privacy (Teng et al., 2022). For example, they might develop discontinuous use behavior, such as taking short breaks, performing information-control activities, or suspending use (Fan et al., 2020).

### 2.3 Theorizing PSW on MSM

Definitions of SW are often context oriented, varying across different situations. In the psychological and behavioral sciences, SW is often considered a specific behavior for coping with stressful situations, such as unwanted social interaction (Evans et al., 2000) and negative experiences (Elison et al., 2006). In organizational management research, SW is mostly limited to work withdrawal and job withdrawal. It is regarded as a way of coping with unfavorable experiences and attitudes in the workplace (Kim et al., 2019) and can involve avoiding or denying communication or cooperation (Xu et al., 2022). Pan (2018) divided withdrawal at work into psychological withdrawal behavior and physical withdrawal behavior; the former refers to remaining physically present at work while mentally escaping from it (Bashir et al., 2019). In addiction research, SW pays more attention to individuals’ subjective feelings than their objective behaviors. It can include negative emotions (Liu et al., 2021), negative moods (Fioravanti and Casale, 2020), and negative feelings (Balakrishnan and Shamim, 2013). In MSM research, SW is mainly a behavior that involves withholding information, mainly to avoid the negative outcomes of communication (Dienlin and Metzger, 2016). It involves avoiding contact with others altogether in addition to avoiding disclosure (Moore and Tangney, 2017). Table 1 reviews the SW-related literature, briefly describing studies’ references, concepts, definitions, contexts, theoretical foundations, focuses, and key findings.

**Table 1:** Studies related with self-withdrawal.

Reference	Concept	Definition	Context	Theoretical foundation	Study focus	Key findings
Evans et al. (2000)	Social Withdrawal	A strategy coping with the difficulty of too much unwanted social interaction.	Chronic Residential Crowding	Systematic Literature Review	Whether the previously documented coping strategy of social withdrawal in response to chronic crowding is related to changes in social information processing.	Individuals residing in crowded homes were less cognizant of personal information about strangers.
Andreassen et al. (2012)	Withdrawal	The occurrence of unpleasant feelings when the activity is discontinued or suddenly reduced.	Facebook Addiction	Systematic Literature Review	Develop Facebook Addiction Scale	The BFAS has acceptable psychometric properties in terms of internal consistency, factor structure, and reliability, as well as in relation to content and convergent and discriminative validity.
Balakrishnan and Shamim (2013)	Withdrawal	Negative feelings experienced when the person is unable to perform the activity.	Facebook	Uses and Gratifications Theory, Brown’s behavioral addiction framework	Facebook usage pattern, motivations and psychological/behavioral factors affecting the users	Malaysian students use Facebook for Social Networking, Psychological Benefits, Entertainment, Self Presentation and Skill Enhancement.
Dienlin and Metzger (2016)	Self-withdrawal	Active retention of information	Facebook	Privacy Calculus	Test the privacy calculus’ generalizability and extend its theoretical framework by including both self-withdrawal behaviors and privacy self-efficacy.	Both privacy concerns and privacy self-efficacy positively predicted self-withdrawal. Regarding self-disclosure, benefits outweighed privacy concerns; regarding self-withdrawal, privacy concerns outweighed both privacy self-efficacy and benefits.
Moore and Tangney (2017)	Social Withdrawal	A form of concealment. It involves avoidance of contact with others altogether in addition to avoidance of disclosure.	Concealable Stigma	Systematic Literature Review	Examine whether criminal offenders’ anticipated stigma predicts behavioral outcomes through social withdrawal.	Anticipated stigma during incarceration predicted social withdrawal three months postrelease, which then predicted more mental health problems 1 year postrelease.
Chen (2018)	Withdrawal Behaviors	Actively deleting and untagging posts	Social Media	Spiral of Silence Theory	The spiral of silence process on social media	Fear of social isolation (FSI) has an indirect effect on discouraging disagreeing opinion expression but not supporting opinion expression and on encouraging withdrawal behaviors through enhancing willingness to self-censor (WTSC) on social media.
Leng et al. (2019)	withdrawal	One of addiction’s core symptoms	Social networking sites addiction	Systematic Literature Review	The effect of different materials on desire and excitability of social network addicts	SNS-related stimulation, especially image clues, could significantly induce subjects for the craving and excitability of social networks, and the craving fluctuates.
Meier et al. (2020)	self-withdrawal	managing and withholding personal information by using privacy protecting software or by using privacy settings.	Facebook	Protection Motivation Theory	Investigate factors that predict Facebook users’ privacy protection motivation and to examine whether fear appeals and social norms can raise participants’ intention to protect personal data	Privacy threat perception and the perceived effectiveness of privacy protection are important factors for explaining self-withdrawal intention among Facebook users.
Fioravanti and Casale (2020)	withdrawal	One of addiction’s core features	Facebook	Systematic Literature Review	Creat and validate a questionnaire to measure active and passive Facebook use, and evaluate its association to Facebook addiction	The APUF is a useful measure with good psychometric properties for assessing whether people use Facebook actively or passively
Ma et al. (2021)	self-withdrawal	Manifested as the withholding of information	WeChat Moments	Privacy Calculus Theory	The influence of ephemerality on the tension between information-privacy concerns and information disclosure	Individuals’ perceptions of the ephemerality of technology features significantly impacted users’ self-disclosure intentions by counteracting perceived severity and perceived intrusion concerning privacy risks.
Li et al. (2022)	withdrawal behavioral intention	The tendency of users to unfollow, leave groups and uninstall platforms.	mainstream social media platforms	cognition-affect-conation framework	Explore how perceived overload affects the passive usage intentions of social media users.	Perceived overload positively affects the passive usage intentions of mobile social media users.

Based on the literature, we can see that SW is a multidimensional concept (Lee, 2021), encompassing both behavioral and psychological dimensions, with the latter often being ignored. Apart from addiction-related studies, the above mentioned studies mostly focus on the behavioral dimension of SW while the psychological dimension is rarely mentioned, especially in MSM research. Although addiction-related studies have paid attention to the psychological dimension, they only focus on the negative feelings that are aroused after activities are ceased, ignoring the negative feelings aroused during activities. However, it is precisely the uncomfortable use process that stimulates users’ bad feelings the most. In addition, the MSM studies that do define SW tend to be Facebook-specific and do not lend themselves to WeChat research. Such definitions are incomplete. China is a strong-tie society where considerable importance is attached to the concept of *guanxi*, which is rooted in Confucianism. WeChat is a social platform on which Chinese people can maintain and enhance *guanxi* with others in their social networks (Zhao and Zhang, 2020). Thus, exploring the psychological dimension of SW on WeChat is particularly meaningful in the Chinese context. As outlined above, we propose the PSW concept and define it as “users’ series of negative feelings when using WeChat.” Then, we develop and validate measurement items for PSW based on our literature review, focus-group interviews, one-on-one in-depth interviews, and a survey.

## 3 Method

Following Hosseini et al. (2022), we employ a mixed-methods design. We derived important information about PSW in the WeChat context using qualitative methods. We then used this information to structure a questionnaire to measure PSW. Qualitative (phase 1) and then quantitative (phase 2) data were collected and analyzed in two consecutive phases. Phase 1 relied on information provided by focus-group and one-on-one in-depth interviews, aiming to form a measurement item pool for PSW. Phase 2 used an online survey to examine the reliability and validity of the PSW measurement items. We created the questionnaire using Sojump. Prior to the questionnaire, respondents viewed an information page describing the purpose of the research and the confidentiality of data.

In the qualitative research, first, we used the focus-group interviews and one-on-one interviews to collect users’ opinions about PSW on WeChat. Then, we used NVIVO 12.0 to analyze the interview data and extract measurement items. An initial item pool was created by combining items collected from the literature review with those extracted from the interviews.

In the quantitative research part, we followed the scale development process of perceived anonymity (Hite et al., 2014) and conducted an online survey based on the initial item pool. SPSS 26.0 was used to carry out exploratory factor analysis (EFA) and confirmatory factor analysis (CFA). This was a multistage process consisting of item selection, expert review, a pretest, pilot testing, and the full testing of the measurement instrument. This method includes numerous evaluations of reliability and validity and offers a rigorous framework for developing a reliable and valid measurement instrument. Throughout the development of the PSW measure, we took the Cronbach’s alpha coefficient as the main indicator of reliability, similar to previous studies (Sigerson and Cheng, 2018; Fioravanti and Casale, 2020). All of the EFA solutions were obtained using maximum likelihood extraction methods and unrotated solutions (similar results were obtained using alpha factoring extraction). During EFA in the pilot studies, factors with eigenvalues greater than one were extracted. CFA solutions executed for a second sample were obtained using maximum likelihood parameter estimation.

**Figure 1:**
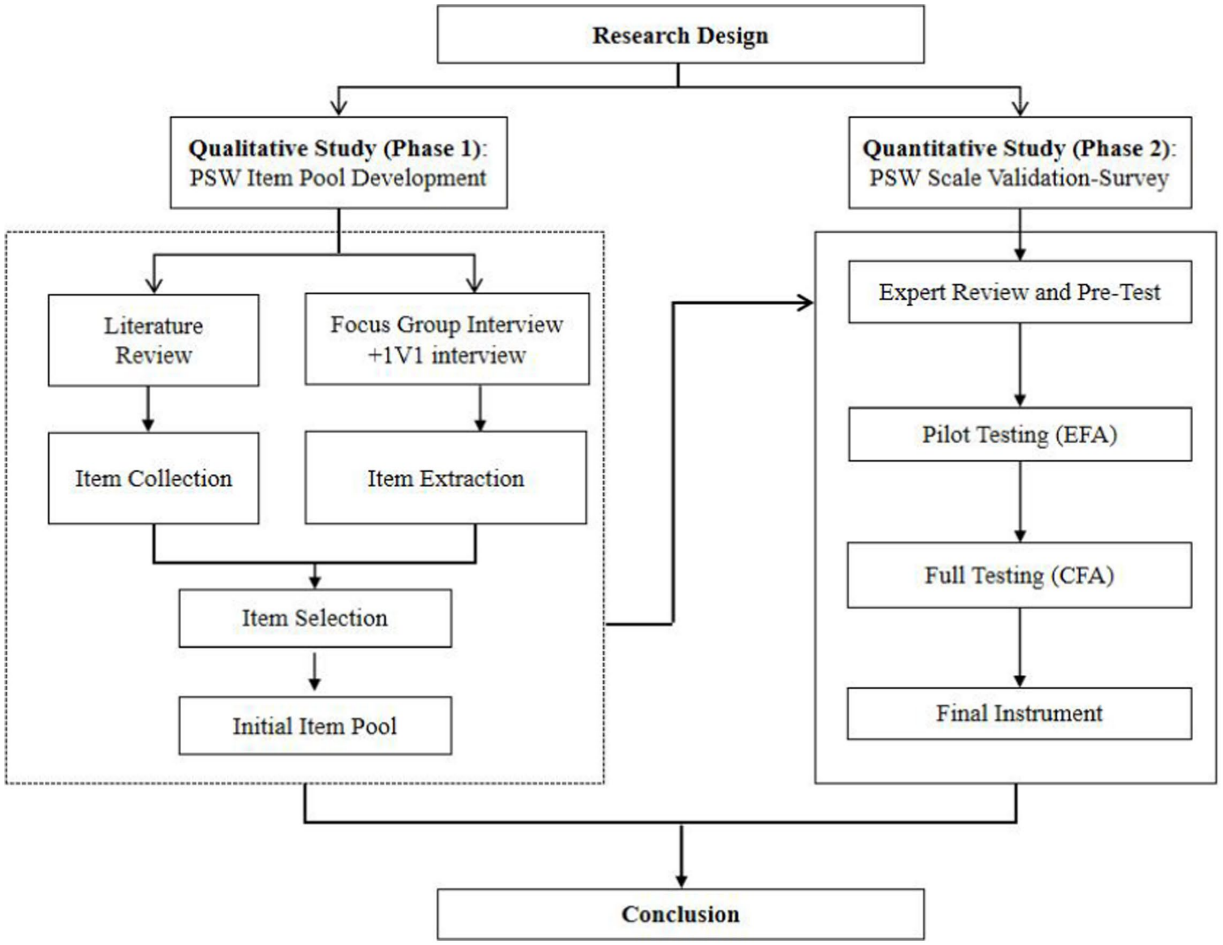
Research design.

The formal measurement items were formed based on the qualitative and quantitative research. Figure 1 shows the research design.

## 4 Phase 1: qualitative research

Based on our literature review, we first collected some measurement items for PSW in the MSM context and revised them to make them suitable for our research. Table 2 shows the revised items. Then, we conducted the focus-group interviews to gain a deeper understanding of PSW.

**Table 2:** Revised measurement items for self-withdrawal.

Measurement items	Sources
I become restless or troubled if I am prohibited from using WeChat	Andreassen et al. (2012)
I often feel timid on WeChat	McDermott et al. (2013)
I limit what I do and say on WeChat	Dienlin and Metzger (2016)
I make my WeChat moments visible only to people I trust	Dienlin and Metzger (2016)
I often delete or withdraw messages or posts on WeChat	Chen (2018)
I delete some friends on WeChat	Li et al. (2022)

### 4.1 Focus-group interview

For the purposes of this study, we defined the PSW construct as “users’ series of negative feelings when using WeChat.” Then, we conducted focus-group interviews to understand respondents’ perceptions of PSW on WeChat. The focus-group format facilitates a dynamic exchange of views (Lopez-Gonzalez et al., 2021). Participants were told that the study involved “exploring negative feelings related to WeChat use that lead to a series of information-control behaviors.”

Focus-group participants needed to meet the following two criteria: (i) they were proficient in using WeChat, and (ii) they had had at least one experience in the past 6 months in which they did not want to reveal information on WeChat. To avoid the possible influence of gender and professional field, we recruited participants from different professional fields, and the gender distribution was equal. In total, eight people participated in the focus group. The participants’ characteristics were as follows: All were recruited from a well-known university in southern China and were professionals in different fields, all had experienced a reluctance to disclose information, and all used WeChat every day. There were four men and four women; they ranged in age from 25 to 40 years old.

The host of the focus group was an MSM researcher who focused on topics related to passive use and had experience with focus-group interviews. The purpose of the focus group was to explore the psychological features that would stop users from self-disclosing on WeChat. Accordingly, we created a discussion guide comprising a series of open-ended questions, as shown in Appendix 1, focused on the following:

1. What does the participant use WeChat for (e.g., information, relationships, and entertainment)?
2. What negative feelings have the participants experienced when using WeChat? (They were asked for examples.)
3. How do the participants use WeChat?
4. Why do the participants use WeChat passively?

The focus-group setting involved the following three steps:

*Step 1*: Before starting the focus-group discussion, the definition of PSW, the purpose of the study, and the rules of the discussion were explained to the participants. Participants were informed of their rights and that they were free to leave the study at any time. They signed consent forms and participated voluntarily. Then, all of the participants were given five minutes to think about their WeChat use experiences.

*Step 2*: Questions were asked based on the discussion guide. The host asked the participants to discuss their own experiences related to PSW on WeChat to reduce subjective biases.

*Step 3*: The host summarized the findings of the discussion and showed them to the participants for follow-ups after the end of the focus-group interviews.

Respondents were encouraged to voice their opinions and were allowed to discuss points with each other. When no new information arose, the focus-group interview was concluded. Then, we conducted a preliminary analysis of the interview recordings, which provided many new ideas for the subsequent one-on-one in-depth interviews. The respondents were asked about their WeChat use habits, including features they typically use. One interviewee said, *My WeChat use is mainly relational use, because it is not very convenient to access information or to be entertained through WeChat*. Interviewees were probed about the feelings they had experienced when using WeChat, such as their concerns about using WeChat. One said, *Once, I commented on a girl’s moments. I took it just as pure comfort. However, my girlfriend saw it and misunderstood it, and then she got angry, which had a bad effect on our relationship. Since then, I have tried not to give likes or comments to others*. Another person said, *Although I have set up groups, the people in the groups cross paths offline, which makes me feel stressed*. When asked about WeChat use patterns (e.g., whether to use WeChat Moment privacy settings or not), even though some interviewees said they had limited some friends’ access to their moments, they still have some worries. For example, one interviewee said, *I want to limit someone’s access to my moments, but it is easy for them to find out about this setting, which would magnify my behavior*. The focus-group session took approximately 2 hours. Some modifications of the discussion guide were then made to be suitable for the one-on-one in-depth interviews.

### 4.2 One-on-one in-depth interviews

To get a better understanding of PSW, we conducted one-on-one in-depth interviews with eight people, using the discussion guide modified based on the focus-group interview results. All of the interviews lasted about 1 hour. We focused on statements related to PSW to illustrate that the interviewees had experienced many uncomfortable feelings in their WeChat use. For example, one interviewee indicated concerns about self-disclosure, stating that *I have to consider so many WeChat friends’ opinions when I want to post on Moment*. Another person reported that *WeChat Moment is a public place for me, and I usually post some scenery-related updates on it; I do not want people who are not familiar with me to know everything about me through my WeChat moments*. Another said, *Sometimes, I worry that I might ignore others’ feelings when I disclose information on WeChat Moment; I also worry that others might misunderstand the statuses I post, so I think it is better to disclose less on Moment*. Similarly, another interviewee noted, *I worry that the updated status will be misunderstood, and misunderstandings can spread, so I might delete what I disclose on WeChat*. Interviewees also revealed their WeChat use stress. For example, one said, *WeChat Moment is a public place, and its nature is different from that of private chats. In a private chat, not replying will be considered impolite behavior, so private chats give others reply pressure*. These examples show that many users have experienced negative feelings when using WeChat.

**Figure 2:**
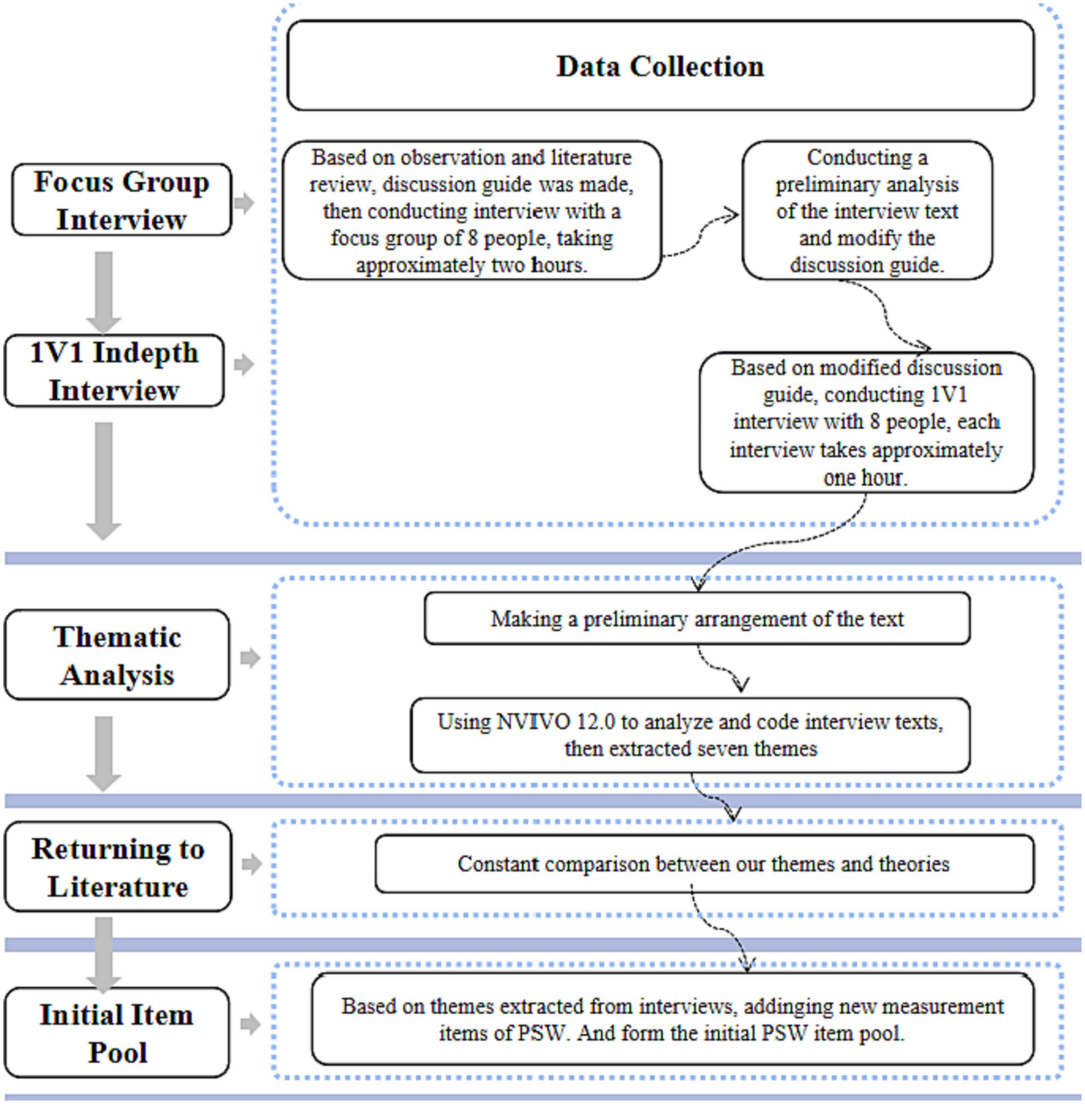
Data collection and analytical approach.

Figure 2 summarizes the main steps we took in the focus-group and one-on-one interviews.

**Figure 3:**
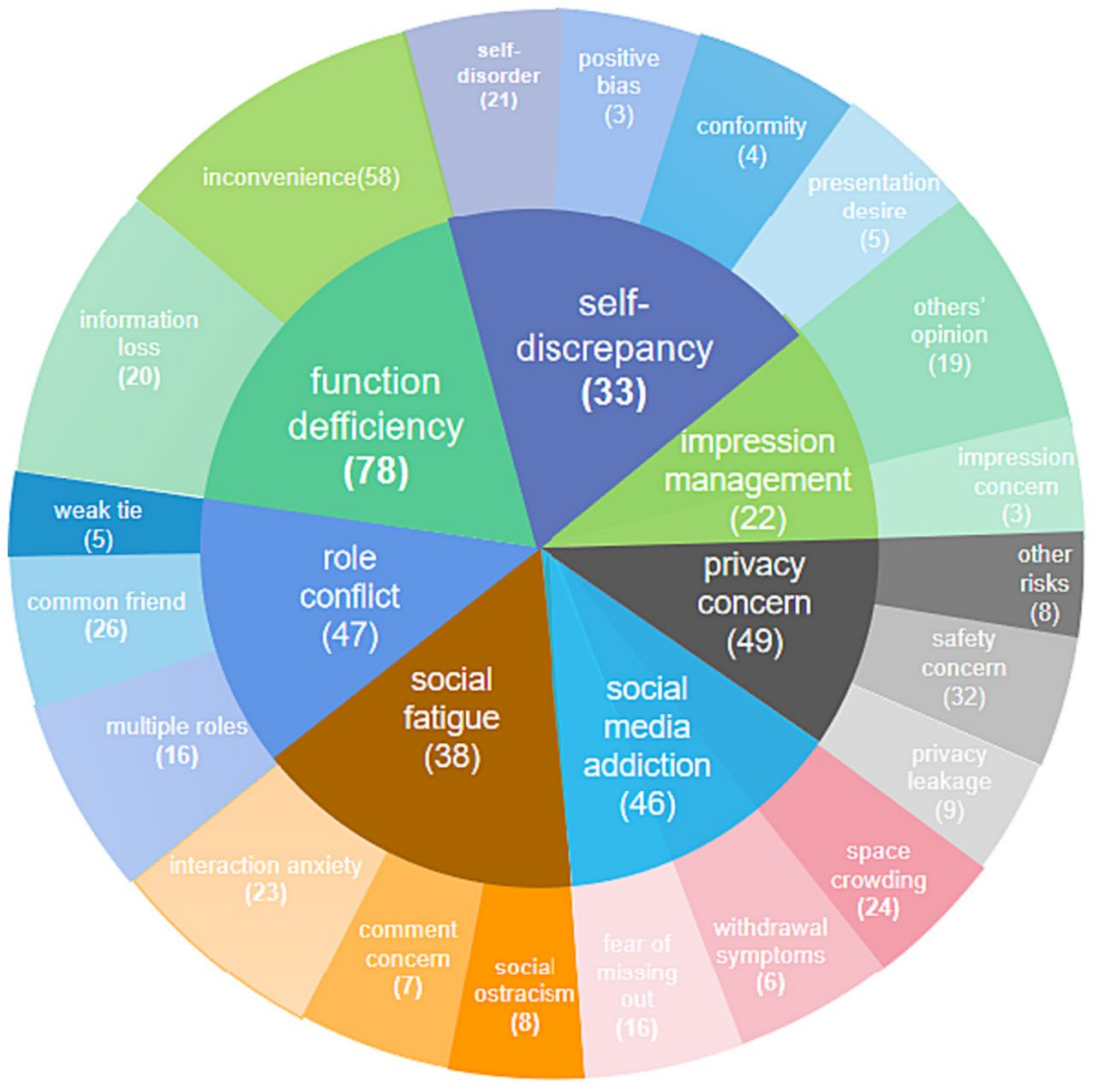
Coding reference point diagram. The number in brackets represents the coding reference point of the variable.

Once the data were collected, we used thematic analysis to identify themes (Lopez-Gonzalez et al., 2021) arising from the focus-group and one-on-one in-depth interviews. We used NVIVO 12 to analyze the content. Following Saldaña (2009), there were two coding cycles. In the first cycle, we divided the data into categories. In the second, we classified, prioritized, integrated, synthesized, abstracted, and conceptualized the content to build a theory. This analysis revealed seven main themes for SW on WeChat: (i) function deficiency, (ii) role conflict, (iii) social fatigue, (iv) social media addiction, (v) privacy concern, (vi) self-discrepancy, and (vii) impression management. Figure 3 shows the coding results, and Table 3 gives examples of the themes.

**Table 3:** Examples of main theme.

Construct	Dimension	Interview example
Psychological Self-withdrawal	Function deficiency	Interviewee: “The main thing is that the storage period of profile information is relatively short, and it will easily expire after a long time”
	Role conflict	Interviewee: “This is similar to normal offline social networking, because one’s connections can include classmates, relatives, friends, leaders, and mentors”
	Social fatigue	Interviewee: “Sometimes I think it is OK if I “like” someone’s moment, but if others comment, I may become confused about whether to reply or not”
	Social media addiction	Interviewee: “In fact, I fear missing out on information; this is more disturbing”
	Privacy concern	Interviewee: “Sometimes, small games require authentication, and I’m a little worried about information leakage”
	Self-discrepancy	Interviewee: “Past posts do not represent who you are now, so of course there’s no need to show them to others”
	Impression management	Interviewee: “Sometimes I post something in a certain emotional state, but when emotion passes, the post may negatively affect my overall image or something, and then I delete it”

According to Liu et al. (2020) and Fan et al. (2020), with increases in users’ social connections on social media, their social circles become more diverse. For example, WeChat friend groups contain acquaintances, friends, relatives, customers, and employees. As a result, WeChat users must play multiple roles and thus experience role conflicts. This makes it difficult to maintain their ideal image, thus leading to lurking behavior, a type of inactive online use behavior. Apart from growing social networks, the functions of WeChat have expanded as well. System function can also be a factor that influences negative MSM use behaviors (Teng et al., 2022). If there are function deficiencies, users might transfer to other platforms. Zhu and Bao (2018) suggested that impression management concern, privacy concern, and social fatigue can all influence passive MSM use. Regarding impression management, self-discrepancy must also be noted. When managing their impression, people struggle with their past self, ideal self, and actual self. If people believe their past disclosure does not represent who they are now, they might experience self-discrepancy. According to self-discrepancy theory, the greater an individual’s self-discrepancy, the more discomfort he or she will suffer, which might result in strategic behaviors adopted to reduce self-discrepancy (Hu et al., 2022). For example, a user might delete his or her past self-disclosure. Self-discrepancy is also likely to be associated with problematic online behavior (Hu et al., 2022), such as WeChat addiction. Hence, based on the themes extracted from interviews, we added new measurement items for SW on WeChat, which are shown in Table 4.

**Table 4:** Measurement items for psychological self-withdrawal extracted from interviews.

Measurement items
I find it inconvenient when I use certain WeChat functions I feel stressed when I play multiple roles on WeChat I feel stressed when I receive interactive messages from others I worry about missing out on information on WeChat
I feel anxious when I disclose personal information on WeChat
I feel uncomfortable when my posts do not represent who I am now
I feel afraid when people see my real but flawed side on WeChat

### 4.3 Item selection

Combining items collected from the literature review with items extracted from the interviews, we developed a 13-item PSW scale (raw scale), as shown in Table 5. In the quantitative research part, we asked respondents to rate these items on a seven-point scale (1 = “strongly disagree” to 7 = “strongly agree”).

**Table 5:** Initial item pool for psychological self-withdrawal.

Construct	Item content	Degree
Psychological Self-withdrawal	I would become restless if I were prohibited from using WeChat I feel timid when I disclose information on WeChat I consciously limit what I do and say on WeChat I make my WeChat moments visible only to people I trust I have deleted or withdrawn my WeChat posts or messages I sometimes delete friends on WeChat I find it inconvenient when I use certain WeChat functions I feel stressed when I play multiple roles on WeChat I feel stressed when I receive interactive messages from others I worry about missing out on information on WeChat I feel anxious when I disclose personal information on WeChat I feel uncomfortable when my posts do not represent who I am now I feel afraid when people see my real but flawed side on WeChat	1 = strongly disagree 2 = very much disagree 3 = disagree 4 = general 5 = agree 6 = very much agree 7 = strongly agree

## 5 Phase 2: quantitative research

### 5.1 Expert review and pretest

The item-construction process for a scale can greatly affect content validity (Sigerson and Cheng, 2018). Therefore, to evaluate the content validity of the PSW measurement instrument, instructors and graduate students at a large university in China reviewed the items. These experts were selected based on their knowledge of self-disclosure, social media, and privacy related to SW. After being given the definition of PSW, they were asked to review the items and provide feedback on how well they represented the inherent aspects of PSW. Additionally, the instrument was pretested using a group of individuals in several course groups. These activities provided important feedback, which prompted minor modifications to the phrasing of some items.

### 5.2 Pilot testing

Two separate pilot tests were used to test reliability and validity, and to refine and enhance the instrument. The first pilot test (Pilot Test 1) involved administering the instrument to a sample of 91 WeChat users. The second pilot test (Pilot Test 2) involved administering the instrument to a subsample of 81 WeChat users taken from the sample used in Pilot Test 1. Participation in the pilot testing was voluntary. Data collected during both pilot tests were subjected to EFA to evaluate the unidimensionality and internal consistency of the instrument.

#### 5.2.1 Pilot Test 1

The initial EFA results obtained from the Pilot Test 1 dataset (Table 6) indicated that the 13 items in the original item pool were dimensional. Four factors with eigenvalues greater than one were extracted. An analysis of the individual item factor loadings indicated that three items (i.e., 1, 3, 4, 5, 6, and 7) had factor loadings lower than 0.6 on all four factors, suggesting poor convergent validity (Fan et al., 2020). Therefore, these items were removed from the item pool to improve the unidimensionality of the instrument, thus reducing the instrument to seven items.

**Table 6:** Pilot Test 1: thirteen psychological self-withdrawal items and maximum likelihood loadings (*n* = 91).

Psychological self-withdrawal item	1	2	3	4
1. I would become restless if I were prohibited from using WeChat	0.327	−0.252	0.242	0.495
2. I feel timid when I disclose information on WeChat	0.686	−0.382	−0.276	−0.189
3. I consciously limit what I do and say on WeChat	0.585	−0.080	0.207	−0.020
4. I make my WeChat moments visible only to people I trust	0.510	0.022	0.449	−0.281
5. I have deleted or withdrawn my WeChat posts or messages	0.394	−0.160	0.352	−0.028
6. I sometimes delete friends on WeChat	0.379	0.508	0.370	−0.080
7. I find it inconvenient when I use certain WeChat functions	0.471	0.054	−0.169	−0.132
8. I feel stressed when I play multiple roles on WeChat	0.704	0.306	−0.015	0.120
9. I feel stressed when I receive interactive messages from others	0.699	0.164	−0.261	−0.011
10. I worry about missing out on information on WeChat	0.616	−0.364	0.217	0.256
11. I feel anxious when I disclose personal information on WeChat	0.746	−0.218	0.112	−0.232
12. I feel uncomfortable when my posts do not represent who I am now	0.710	0.111	−0.192	0.307
13. I feel afraid when people see my real but flawed side on WeChat	0.769	0.324	−0.115	−0.015

The removal of item 1 was justified in that this item did not relate to users’ experience of using WeChat. Item 1 states, “I would become restless if I were prohibited from using WeChat.” This item loaded relatively heavily onto the fourth principal component, which was likely associated with WeChat nonuse experience. The removal of items 3, 4, 5, and 6 was also theoretically justified in that all of these items were related to the consequences of SW. Item 3 states, “I consciously limit what I do and say on WeChat.” Item 4 states, “I make my WeChat moments visible only to people I trust.” Item 5 states, “I have deleted or withdrawn my WeChat posts or messages.” Item 6 states, “I sometimes delete friends on WeChat.” Item 7 states, “I find it inconvenient when I use certain WeChat functions.” This item is related to environmental stimuli and can be considered an antecedent of internal PSW.

The results obtained from the EFA of the remaining seven items (Table 7) indicated the unidimensionality of the instrument. In general, the removal of the aforementioned items provided acceptable factor loadings for each individual item onto a single factor. However, an analysis of the individual item factor loadings indicated that there was still an item (i.e., 10) with a factor loading below 0.6 on the single factor. This can also be theoretically justified in that the fear of missing out is considered a type of social anxiety (Li et al., 2022), and high levels of anxiety can lead social media users to withdraw from Internet activities (Swiatek et al., 2021). Therefore, this item is related to the antecedent of PSW and was removed from the item pool to improve the reliability of the measurement instrument, thus reducing the instrument to six items.

**Table 7:** Pilot Test 1: seven psychological self-withdrawal items and maximum likelihood loadings (*n* = 91).

Psychological self-withdrawal item	1
2. I feel timid when I disclose information on WeChat	0.630
8. I feel stressed when I play multiple roles on WeChat	0.728
9. I feel stressed when I receive interactive messages from others	0.733
10. I worry about missing out on information on WeChat	0.526
11. I feel anxious when I disclose personal information on WeChat	0.652
12. I feel uncomfortable when my posts do not represent who I am now	0.746
13. I feel afraid when people see my real but flawed side on WeChat	0.801

The results obtained from the EFA of the remaining six items (Table 8) indicated the unidimensionality of the instrument. Furthermore, the removal of the aforementioned item provided acceptable factor loadings for each individual item onto a single factor. The remaining six items explained 51.22% of the total variance. The reliability coefficient for the resulting six-item instrument was 0.861 in Pilot Test 1.

**Table 8:** Pilot Test 1: six psychological self-withdrawal items and maximum likelihood loadings (*n* = 91).

Psychological self-withdrawal item	1
2. I feel timid when I disclose information on WeChat	0.607
8. I feel stressed when I play multiple roles on WeChat	0.736
9. I feel stressed when I receive interactive messages from others	0.750
11. I feel anxious when I disclose personal information on WeChat	0.619
12. I feel uncomfortable when my posts do not represent who I am now	0.736
13. I feel afraid when people see my real but flawed side on WeChat	0.821

#### 5.2.2 Pilot Test 2

The second pilot test included the EFA of the refined six-item instrument obtained from Pilot Test 1, using the subsample of 81 participants taken from the sample used in Pilot Test 1. The analysis results (Table 9) showed that only a single factor with an eigenvalue over one was extracted, and this factor explained 50.85% of the total variance. However, an analysis of individual item factor loadings indicated that two items (i.e., 12 and 13) exhibited factor loadings below 0.6 on the factor. Therefore, these items were removed from the item pool to improve the unidimensionality of the instrument, thus reducing the instrument to four items.

**Table 9:** Pilot Test 2: six psychological self-withdrawal items and maximum likelihood loadings (*n* = 81).

Psychological self-withdrawal item	1
2. I feel timid when I disclose information on WeChat	0.882
8. I feel stressed when I play multiple roles on WeChat	0.813
9. I feel stressed when I receive interactive messages from others	0.671
11. I feel anxious when I disclose personal information on WeChat	0.833
12. I feel uncomfortable when my posts do not represent who I am now	0.441
13. I feel afraid when people see my real but flawed side on WeChat	0.524

The removal of item 12 was justified in that some users use WeChat as a work platform and rarely update posts; thus, they lack such experience. This motivation type can be considered an antecedent of PSW. Item 12 states, “I feel uncomfortable when my posts do not represent who I am now.” This item loaded relatively lowly onto the principal component. The removal of item 13 was also justified in that this item is related to the antecedents of PSW. Item 13 states, “I feel afraid when people see my real but flawed side on WeChat.”

The results obtained from the EFA of the remaining four items (Table 10) indicated the unidimensionality of the instrument. Furthermore, the removal of the aforementioned items provided acceptable factor loadings for each individual item onto a single factor. The remaining four items explained 64.40% of the total variance. Each individual item loaded well onto the single factor. The reliability coefficient for the resulting four-item instrument was 0.876 in Pilot Test 2.

**Table 10:** Pilot Test 2: four psychological self-withdrawal items and maximum likelihood loadings (*n* = 81).

Psychological self-withdrawal item	1
2. I feel timid when I disclose information on WeChat	0.907
8. I feel stressed when I play multiple roles on WeChat	0.788
9. I feel stressed when I receive interactive messages from others	0.640
11. I feel anxious when I disclose personal information on WeChat	0.850

### 5.3 Full testing

The full testing of the instrument involved collecting responses from 1,200 participants using an online platform that provides sample collection services for a fee. Participants completed an online survey including the PSW instrument. Participants accessed the survey via a QR code. Table 11 shows the demographic data.

**Table 11:** Demographics characteristics of participants.

Variables	Level	Frequency	Percentage (%)
Gender	Male	617	51.42
	Female	583	48.58
Education	High school and under	152	12.67
	Junior college or Bachelor	869	72.42
	Master	141	11.75
	Doctor	38	3.17
Length of WeChat use (days)	Less than 2 h	330	27.5
	2–3 h	452	37.67
	3–4 h	258	21.50
	Over 4 h	160	13.33
Number of WeChat friends	Less than 300	354	29.50
	301–1,000	472	39.33
	1,001–2000	306	25.50
	over 2000	68	5.67

**Figure 4:**
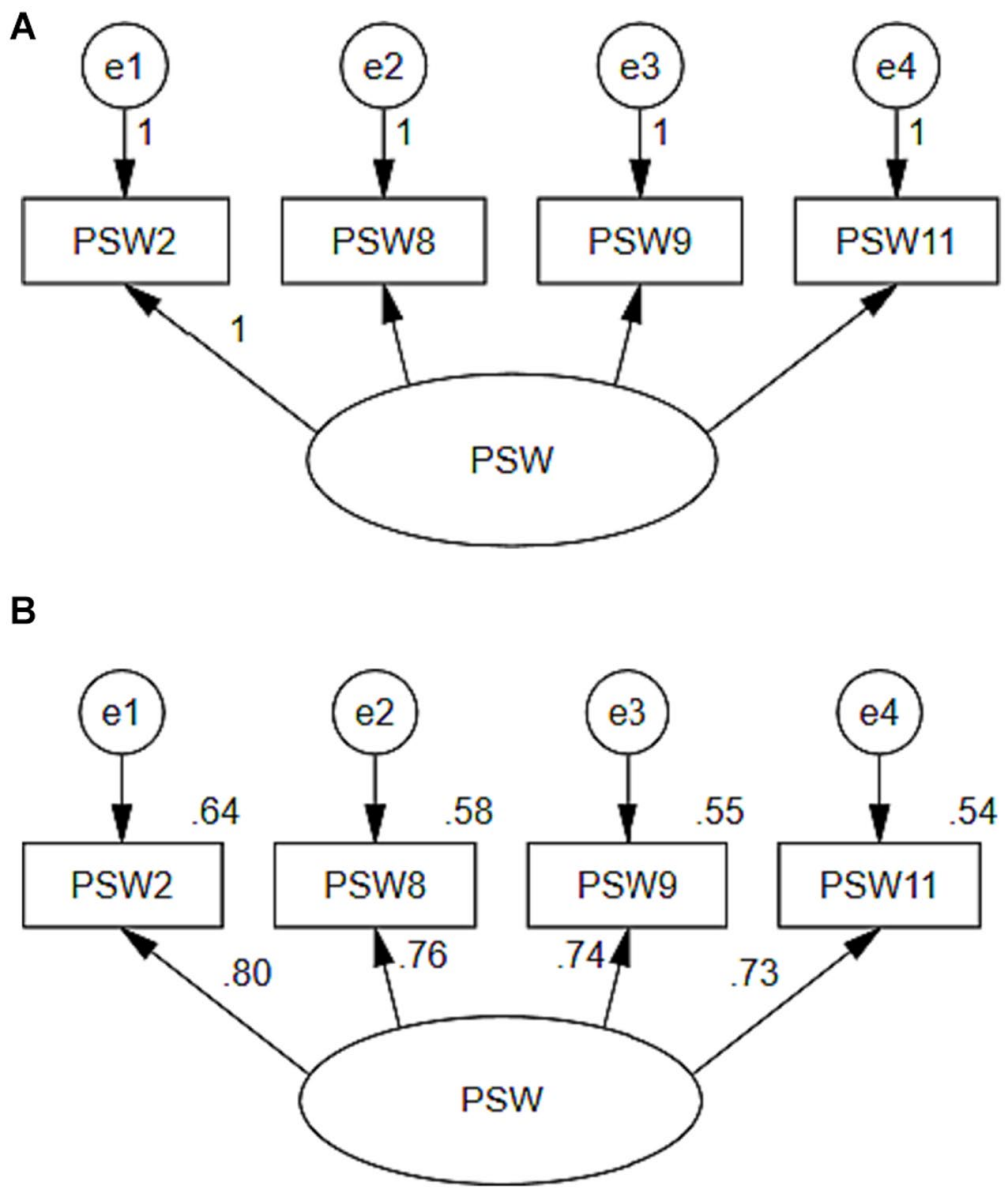
**(A)** Psychological self-withdrawal CFA model for data set A (*n* = 600). See Table 10 for the psychological self-withdrawal items. The results of this analysis indicated that the model adequately fit the data compared with the independence model. The comparative fit indices (NFI = 0.995, NNFI/TLI = 0.992, CFI = 0.997) were above the generally accepted level of 0.95. The chi-square/degrees-of-freedom ratio (*χ*^2^/df) was 2.126, which is below the generally accepted level of 3. RMSEA was 0.043, which is below the generally accepted level of 0.05 (Ma et al., 2021). These results indicated that a single-factor model including items 2, 8, 9, and 11 provided an excellent fit to the data. **(B)** Psychological Self-withdrawal CFA model for data set B (*n* = 600). See Table 10 for the psychological self-withdrawal items. Standardized maximum likelihood parameter estimates shown. All parameter estimates are significant at the *p* < 0.001 level.

The full testing of the instrument included CFA using the full test dataset. To allow for thorough testing, and because the full test dataset was sufficiently large, the dataset was randomly split into two sets (i.e., sets A and B). CFA was first conducted on Model A (Figure 4A) using dataset A.

Model A was renamed Model B (Figure 4B) and used to evaluate the model using dataset B (independent from dataset A), and a separate CFA was conducted. Model B had excellent comparative fit indices (NFI = 0.999, NNFI/TLI = 1.005, CFI = 1.000), which were also above the generally accepted level of 0.95. The chi-square/degrees-of-freedom ratio (χ^2^/df) was 0.275, which is below the generally accepted level of 3. RMSEA was 0.000, which is below the generally accepted level of 0.05 (Ma et al., 2021). While there was overfitting, studies have verified that a model with excellent fit indices (NNFI/TLI > 1, CFI = 1, RMSEA = 0) is also good and acceptable, whether it is a large-sample model or a small-sample model (Cooper et al., 2008; Audrain-McGovern et al., 2009). Thus, these results also indicated that a single-factor model including items 2, 8, 9, and 11 provided an excellent fit with the data. Hence, the final PSW measurement instrument consisted of these four items.

## 6 Discussion

We define PSW as “users’ series of negative feelings when using WeChat” and develop and validate an PSW measurement, using focus groups, interviews, and a survey. A four-item PSW with acceptable reliability and validity was finally developed. The results of the pilot tests and final instrument development provide convincing evidence for the reliability and validity of the new PSW measure in the WeChat context. Appendix 2 presents the final instrument.

The reliability and validity of the instrument were evaluated throughout its development. The reliability coefficients obtained during pilot testing were at least 0.850, and the reliability coefficient for the full test was 0.831. These reliability coefficients are well above 0.7, which is the generally accepted value (Sigerson and Cheng, 2018). Thus, there is evidence that the PSW instrument presented here has sufficient reliability. The instrument’s content validity was established during item selection, expert review, and pretesting. The extraction of a single factor with an eigenvalue greater than one during pilot testing established unidimensionality, as did the CFA results obtained during full testing, which evaluated construct validity. All of these evaluations and the subsequent results provide evidence that the PSW instrument has sufficient validity.

Among the four measurement items of PSW, “I feel timid when I disclose information on WeChat” was found to have the highest factor loading (0.907), while “I feel anxious when I disclose personal information on WeChat” had the second-highest factor loading (0.850). This indicates that WeChat users experience strong psychological withdrawal before disclosing information. “I feel stressed when I play multiple roles on WeChat” had the third-highest factor loading (0.788), while “I feel stressed when I receive interactive messages from others” had the lowest factor loading (0.640). If users experience strong role conflicts and social interaction anxiety when using MSM, they will develop physical withdrawal behavior. This aligns with previous studies (Fan et al., 2020; Liu et al., 2020).

### 6.1 Theoretical implications

Previous scales and definitions were mostly limited to Facebook and focused on the behavioral dimension. Our study, therefore, deliberately defines and measures PSW on WeChat in the psychological dimension. This makes our scale suitable for measuring PSW on strong-tie platforms. Use behavior on WeChat is different from that of other MSM platforms (Chen et al., 2019). There is a need, then, for a relatively particular PSW instrument that can be used in the WeChat context. This study can provide a reference for future instrument development, allowing for modifications of other MSM contexts to customize the instrument for a particular context. The relationships between PSW on MSM and its antecedents and consequences are still not fully understood. This study takes a first step toward advancing our understanding of such relationships by defining PSW and providing researchers with a reliable and valid instrument for measuring PSW in the WeChat context. Given that such an instrument was previously nonexistent, this study makes a meaningful contribution to the field.

### 6.2 Managerial implications

Studying the PSW among MSM users has significant implications for platform enterprises like WeChat. When users experience PSW, they may become less likely to interact with other users or spend less time on the platform, ultimately leading to a decrease in user engagement and revenue. As such, it is crucial for platform enterprises like WeChat to understand and address PSW among their users. One suggestion to reduce PSW is to improve the user experience by enhancing platform design and features, such as providing personalized recommendations or creating a more intuitive user interface. Additionally, platform enterprises can facilitate communication and engagement among users to foster a sense of community and belonging, which may further reduce PSW. Ultimately, addressing PSW is essential for platform enterprises like WeChat to remain relevant and competitive in the ever-evolving social media landscape.

### 6.3 Limitations and future research

In an attempt to develop an PSW measurement instrument for an acquaintance MSM platform, we reviewed the literature and designed interview questions to explore WeChat users’ opinions about PSW. The use of convenient student samples during the pilot testing might have limited the results.

Given that few studies have considered PSW on MSM, there are many possibilities for future research. Future studies could, for example, test the reliability and validity of the PSW instrument in other contexts, such as ZhiHu, QQ, and Weibo. An interesting possibility is the replication of previous SW studies where, instead of treating SW as simply a negative MSM use phenomenon, researchers could capture the relationship between psychological SW and behavioral SW. Longitudinal studies involving SW could also be promising; for example, we could explore the antecedents and consequences of SW on different MSM platforms. It would also be interesting to evaluate whether psychological SW will lead to behavioral withdrawal.

## 7 Conclusion

In this study, we introduce and evaluate psychological self-withdrawal as users’ series of negative feelings when using WeChat. Then we develop and validate scales to measure psychological self-withdrawal using mixed methods, involving interview and survey. Given the flourishing development of research on mobile social media in recent years, mobile social media has become an essential part of people’s lives, and the self-withdrawal of mobile social media users is an important research area that needs further development. Although there are many promising scales, each scale focuses on behavioral withdrawal. This study can provide effective guidance for researchers planning to conduct MSM withdrawal research and may provide new psychological measurement research for further evaluating scales of self-withdrawal among MSM users.

## Data availability statement

The raw data supporting the conclusions of this article will be made available by the authors, without undue reservation.

## Ethics statement

Ethical approval was not required for the studies involving humans because according to the law and related policies, our present research does not require ethical approval. The studies were conducted in accordance with the local legislation and institutional requirements. The participants provided their written informed consent to participate in this study.

## Author contributions

CC: Conceptualization, Data curation, Formal analysis, Investigation, Methodology, Software, Supervision, Validation, Writing – original draft, Writing – review & editing. HY: Data curation, Formal analysis, Funding acquisition, Methodology, Software, Validation, Writing – review & editing, Resources.

## Appendix A

**Focus-group discussion guide**

1. Do you have any memorable experiences that cause you to feel uncomfortable when using WeChat?
2. Do you have any concerns when using WeChat’s various functions?
3. When using WeChat, have you ever experienced a situation in which you want to block certain people but dare not block them? Why would this happen?
4. When using WeChat, do you ever experience a situation in which you want to post a moment but dare not post? Why would this happen?
5. When using WeChat, have you ever made a post and then deleted it or sent a message and later withdrew it? Why would you do that?
6. Are you familiar with WeChat’s privacy settings? Which privacy settings have you used?
7. Would you stop using WeChat if you could? Why?
8. In addition to the above problems, do you have anything to add about self-withdrawal on WeChat?

## Appendix B

**Final psychological self-withdrawal measurement instrument** To what extent do you agree with the following statements related to the negative use behaviors of WeChat users?

I feel timid when I disclose information on WeChat.
I feel stressed when I play multiple roles on WeChat.
I feel stressed when I receive interactive messages from others.
I feel anxious when I disclose personal information on WeChat.

<Responses range from strongly disagree to strongly agree on seven-point Likert scales.>.
